# Virtual Treatment Zone From Cone Beam CT Commonly Alters Treatment Plan and Identifies Tumor at Risk for Under-Treatment in US or US Fusion-Guided Microwave Ablation of Liver Tumors

**DOI:** 10.1177/15330338231181284

**Published:** 2023-08-22

**Authors:** Antonio Arrichiello, Anna Maria Ierardi, Alessandro Caruso, Pasquale Grillo, Letizia Di Meglio, Pierpaolo Biondetti, Massimo Iavarone, Angelo Sangiovanni, Salvatore Alessio Angileri, Chiara Floridi, Bradford Wood, Gianpaolo Carrafiello

**Affiliations:** 1 Operative Unit of Radiology, Fondazione IRCCS Ca’ Granda Ospedale Maggiore Policlinico di Milano, Milan, Italy; 2 Center for Interventional Oncology, Radiology and Imaging Sciences, Clinical Center, National Institutes of Health, Bethesda, MD, USA; 3 SC Gastroenterology and Hepatology, Fondazione IRCCS Ca’ Granda Ospedale Maggiore Policlinico di Milano, Milano, Italy; 4 Department of Clinical, Special and Dental Sciences, Division of Interventional Radiology, University Politecnica delle Marche, Ancona, Italy; 5 Department of Health Sciences, 9304 Università degli Studi di Milano, Milano, Italy

**Keywords:** 4D CBCT, ablation, cancer, liver cancer, radiology, intraoperative imaging

## Abstract

Tumor ablation is included in several major cancer therapy guidelines. One technical challenge of percutaneous ablation is targeting and verification of complete treatment, which is prone to operator variabilities and human imperfections and are directly related to successful outcomes, risk for residual unablated tumor and local progression. The use of “Prediction Ablation Volume Software” may help the operating Interventional Radiologist to better plan, deliver, and verify before the ablation, via virtual treatment zones fused to target tumor. Fused and superimposed images provide 3-dimensional information from different timepoints, just when that information is most useful. The aim of this study is to evaluate the technical success and efficacy of an ablation treatment flowchart provided by a cone beam computed tomography (CBCT) “Prediction Ablation Volume Software.” This is a single-center retrospective study. From April 2021 to January 2022, 29 nonconsecutive evaluable patients with 32 lesions underwent liver ablation with Prediction Ablation Volume Software. Each patient was discussed in a multidisciplinary tumor board and underwent an enhanced computed tomography or magnetic resonance imaging approximately 1 month before the procedure, as well as ∼1 month after. Technical success was defined as treatment of the tumor according to the protocol, covered completely by the Prediction Ablation Volume. Technical efficacy was defined as assessment of complete ablation of the target tumor at imaging follow up (∼1 month). Technical success, technical efficacy, and procedural factors were studied. Technical success was achieved in 30 of 32 liver lesions (94%), measuring 20 mm mean maximum diameter. The antenna was repositioned in 16 of 30 (53%) evaluable target lesions. Residual tumor was detected at 1 month imaging follow up in only 4 of 30 (13%) of the treated lesion. Technical efficacy was of 87% in this retrospective description of our process. The implementation of a CBCT Prediction Ablation Volume Software and flowchart for the treatment of liver malignancies altered the procedure, and demonstrated high technical success and efficacy. Such tools are potentially useful for procedural prediction and verification of ablation.

## Introduction

The interventional oncology (IO) is a highly specialized practice within interventional radiology (IR) that gained an increasingly crucial role in the treatment of certain local or oligometastatic cancers. Its first applications started in the early 1970s, with endovascular infusion of chemotropic drugs that evolved years later in the modern endovascular treatments such as transarterial chemoembolization, transarterial radioembolization, and transarterial embolization.^1^ Subsequently, in the 1980s, first liver percutaneous therapies were performed, consisting of ultrasound (US)-guided hepatocellular carcinoma (HCC) percutaneous ethanol injection.^2^ This procedure opened the field to the modern needle-based ablation techniques performed nowadays, using novel ablation technologies. Percutaneous approaches include different ablation technologies such as radiofrequency, microwaves (MW), cryoablation laser, chemoporation, and irreversible electroporation.^3,4^ IO has evolved rapidly in recent years becoming a dynamic, innovative, fast growing, and minimally invasive approach to cancer treatment. Year after year new technologies helped interventional radiologists to improve the outcomes of patients, with the development of new imaging guidance, software, and devices.^5^ Thanks to these key developments, ablation treatments are now included in multiple major cancer treatment guidelines. Ablation of liver lesions is recommended for HCC treatment by the european association for the study of the liver (EASL) and by Barcelona Clinic Liver Cancer (BCLC);^6,7^ for liver metastasis from colorectal cancer (CRC) by the european society for medical oncology (ESMO) guidelines.^8^ Moreover percutaneous ablation of the liver may be considered in selected patients with other primary tumors (intrahepatic cholangiocarcinoma) or secondary tumors from non-CRC (neuroendocrine, breast, thyroid cancer, melanoma and in oligometastatic disease controlled with systemic treatment).^9^^,^^10^ Percutaneous ablation is less invasive, more cost-effective, and may be repeated easier than surgery.^11,12^ Many of the main challenges and disadvantages of percutaneous in situ ablation are related to the risks for leaving behind residual unablated tumor cells and for local progression, often due to less-than-perfect targeting the lesion.^13^ These limitations are well-described in literature.^14–18^ There are several articles evaluating margin ablation, since it seems to be one of the most important factors associated with local progression that may sometimes be addressed and controlled by the interventional radiologist while performing the procedure.^19,20^ One technical challenge of percutaneous ablation is targeting, navigation, and verification of complete treatment, which is prone to operator variabilities and human imperfections. Optimal targeting, navigation, and verification are directly related to successful outcomes, and risk for residual unablated tumor and local progression result from imperfections, some of which may be mitigated by tools for automation, standardization, and better use of the 3-dimensional (3D) information from software that best layers and displays the 3D imaging data from our exquisite imaging tools. Accurate margin ablation has been reported to be associated with lower local recurrence and is recommended by CIRSE Standard of Practice.^21^ Residual unablated tumor and local progression may be reduced by real-time evaluation of the ablation and its margin, which may be best performed via fusion imaging and verification. Based on this concept, the use of “Prediction Ablation Volume Software” may help interventional radiologists to perform procedures and plan, implement, and verify complete coverage of the target lesions with margin before, during, and after the ablation, all within the fusion flowchart.

## Aim

Evaluate the technical success and efficacy of an ablation treatment flowchart provided by a cone beam computed tomography (CBCT) “Prediction Ablation Volume Software.”

## Material and Methods

This is a single-center retrospective descriptive study. The Institutional Review Board does not require ethical approval for descriptive study. Informed consent for the procedure was obtained from all patients before the intervention. The reporting of this study conforms to STROBE guidelines.^22^

### Patients

Data from 29 nonconsecutive patients were retrospectively evaluated from a prospective tumor ablation registry approved by the internal review board. Eligible patients were treated for liver tumors using the Prediction Ablation Software treatment flowchart between April 2021 and January 2022. Patient treated without this technology were excluded. The nonconsecutive selection was obligatory since the use of the software was not able to be performed by all operators in our practice. Each patient treated in our institution was discussed by a multidisciplinary tumor board (MDT) formed by interventional radiologists, hepatologists, surgeons, and nuclear medicine physicians. The MDT suggested percutaneous ablation of the lesions based on guidelines, disease stage, age, comorbidity, and coagulation status. A contrast computed tomography (CT) or magnetic resonance imaging (MRI) was performed approximately within 1 month before and after the procedure in all 29 patients.

Thirty-two liver malignancies were treated:

- 28 HCC lesions (Lirads 4-5^23^ in 25 patients, histologically proved in 3);
- 3 CRC metastasis (histological diagnosis);
- 1 hepatocholangiocarcinoma (HCC-CC, histological diagnosis).

The average tumor maximum diameter of the selected lesions was 20 mm (range 11-36 mm), and the average tumor volume was 2,2cm^3^ (0.6-4.7 cm^3^).

The population data are summarized in Table 1 and in Supplemental Table a.

**Table 1.** The Population's Data are Summarized.

Total patients	29
Age mean (range) years	70 (46-85)
Gender, n(%)	
Male	25(86)
Female	4(14)
Total tumor	32
Patients with 2 lesions	3
Tumor size mean (range) mm	20 (11-36)
Tumor volume mean (range) cm^3^	2.2 (0.6-4.7)
Tumor type	
HCC (radiological)	25
HCC histology	3
METS histology	3
Hepatocholangiocarcinoma histology	1

Abbreviation: HCC, hepatocellular carcinoma; METS, metastasis.

### Angio-Suite

Every procedure was performed in our Angio-Suite (Azurion ClarityIQ, Philips Healthcare), equipped with a dedicated workstation, CBCT software (XperGuide System, Philips), and US (EPIQ 5 Elite, Philips Healthcare) with US fusion software (PercuNav, Philips). PercuNav fusion software allows targeting lesions which aren’t detectable with US imaging, but are seen on specific phases or sequences in CT, MRI, CBCT, or positron emission tomography (PET). ^24^

### Preprocedure

Every patient was re-evaluated immediately before the procedure by the hepatologist, interventional radiologist and the anesthesiologists to confirm the eligibility to the intervention (assessing onset of new comorbidities, change in coagulation-status, correct interruption of anticoagulant therapies, etc).^25^ Preprocedure intravenous antibiotic prophylaxis with 2 g of cefazolin (Cefazolina Teva, Teva Italia S.r.l.) was administrated to each patient.

Before the ablation, the contrast-CT or MRI were uploaded on the dedicated workstation of the angio-suite. Then a segmentation of the hepatic lesions was semi-automatically executed with assistance from the segmentation system (Segmentation Tool, Philips). In 7 cases the lesions weren’t detectable with US alone, and the segmentation was performed also on the US (EPIQ 5 Elite, Philips) to allow fusion US-CT or US-MRI to target the lesion using the PercuNav software.

### Procedure Flowchart

**Figure 1.**
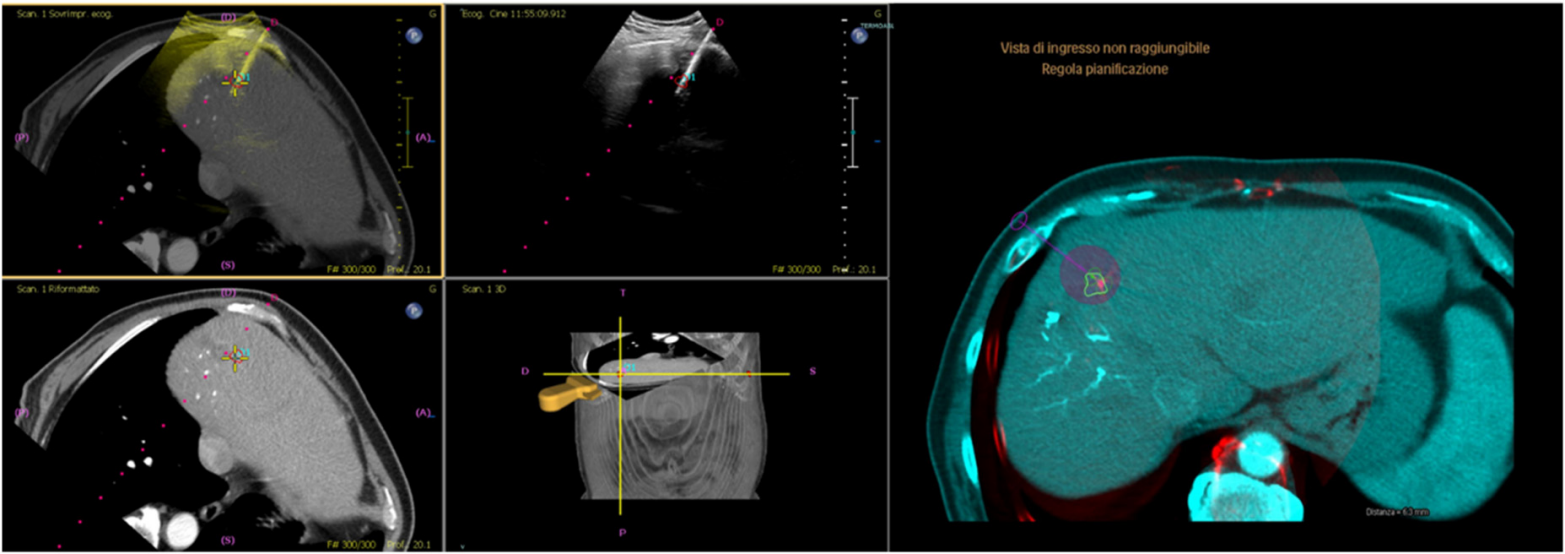
Fusion and CBCT images for a patient with HCC treated with both CBCT fusion and US-CT fusion. Navigation and probe placement were via Percunav Software fusion. After the placement, the correct position is confirmed with CBCT-CT fusion and Volume Ablation Prediction Software was then used (purple) to evaluate and verify adequate margin ablation of the segmented lesion (green). Abbreviations: CBCT, cone beam computed tomography; CT, computed tomography; HCC, hepatocellular carcinoma; US, ultrasound.

**Figure 2.**
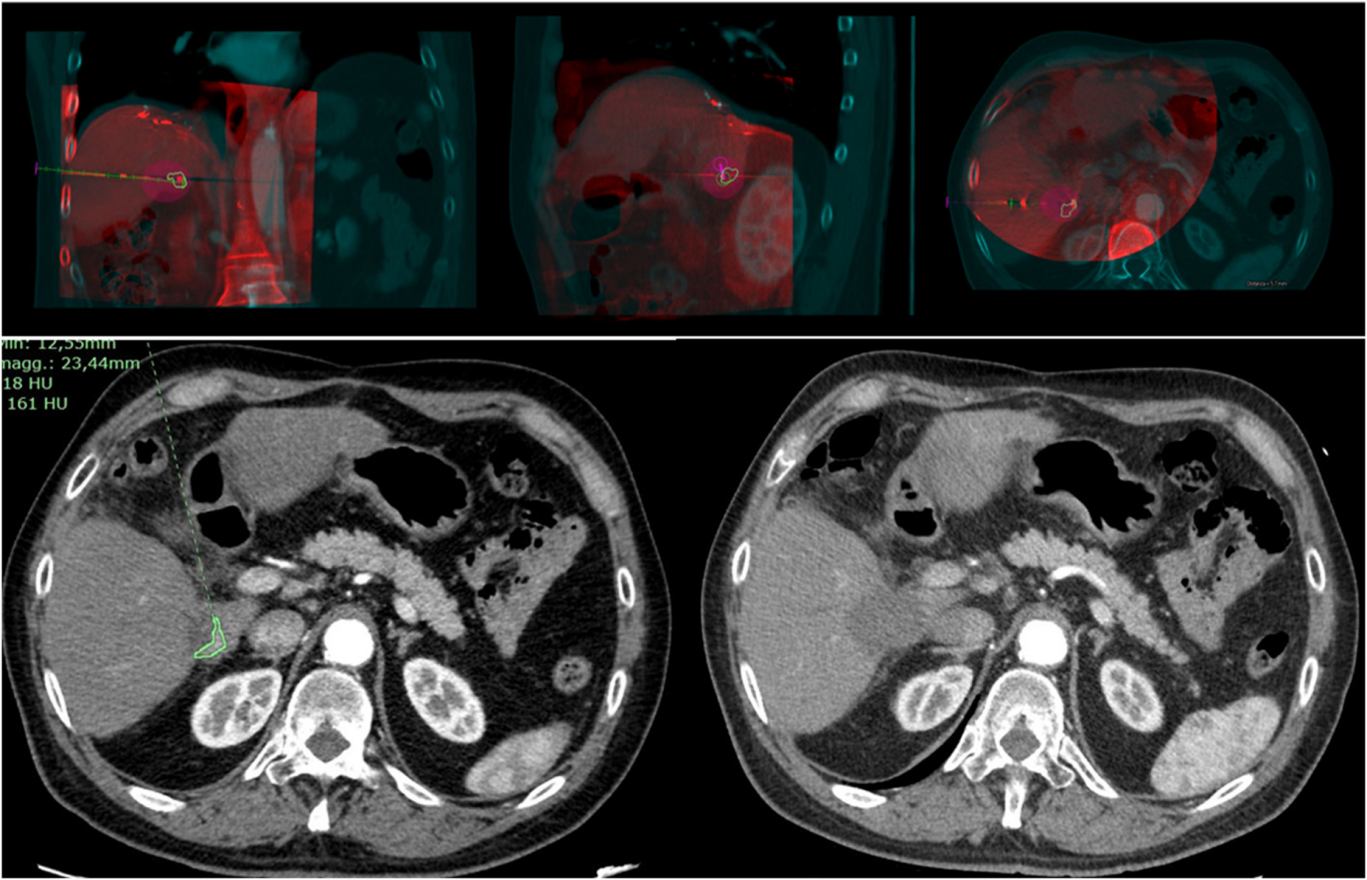
The first 3 images are CBCT + CT fusion (coronal, sagittal, axial) after antenna placement. The Volume Prediction (purple) shows adequate ablative margin. The images are 2 arterial axial CT scans: the first is before the procedure, with lesion segmentation (green) and the second CT is the FU, with complete ablation of the lesion as predicted by volume ablation software. Abbreviations: CBCT, cone beam computed tomography; CT, computed tomography; FU, follow up.

All the procedure session were performed under local anesthesia and moderate sedation with anesthesiology assistance and monitoring. All the ablations were performed using Emprint™ Microwave Ablation System with Thermosphere™ Technology (Medtronic-Covidien). The procedures were performed by interventional radiologists with at least 5 years of experience. Once the desired probe placement was obtained, a noncontrast CBCT was performed during expiration and apnea, like previously described.^26^ The XperGuide System allowed the manual rigid fusion of the previous CT/MRI, where the lesion was segmented, with the CBCT. Elastic fusion is not available in our workstation, but we think that rigid fusion may help emphasizing nearby liver anatomy, and does not deform the data. Once the images were correctly merged, the location of the antenna was evaluated. On the obtained fusion imaging, using the XperGuide System, a virtual probe was overlapped to the antenna. Then selecting a determinate power (W) and time (min) the prediction software automatically created a superimposed visual ablation volume based on the data provided by the producer of the device (Figures 1 and 2). The ablation volume can be assessed in real-time changing the power and time of the ablation to obtain optimal visual coverage of the segmented lesion with appropriate margin. Moreover the presence of neighboring visible nontarget anatomy was preserved. The probe placement was evaluated judging the CBCT-CT/MR fusion and assessing the complete coverage of the segmented lesion and its margin with the virtual ablation spheres. If the coverage was inadequate, the antenna was repositioned to avoid undertreatment and a new CBCT was acquired and re-evaluated. Once final desired probe position was achieved, the ablation was performed according to manufacturer's guidelines.

The ablation protocol power was constant at 100 W in every treatment with variable times adjusted to obtain a minimum ablative margin of 5 mm for each lesion (detailed in Supplemental Table b). After the ablation, a noncontrast CBCT or sequential US controls are performed to assess for potential complications. A ∼1 month follow up (FU) contrast CT or MRI was performed in all the treated patients.

**Figure 3.**
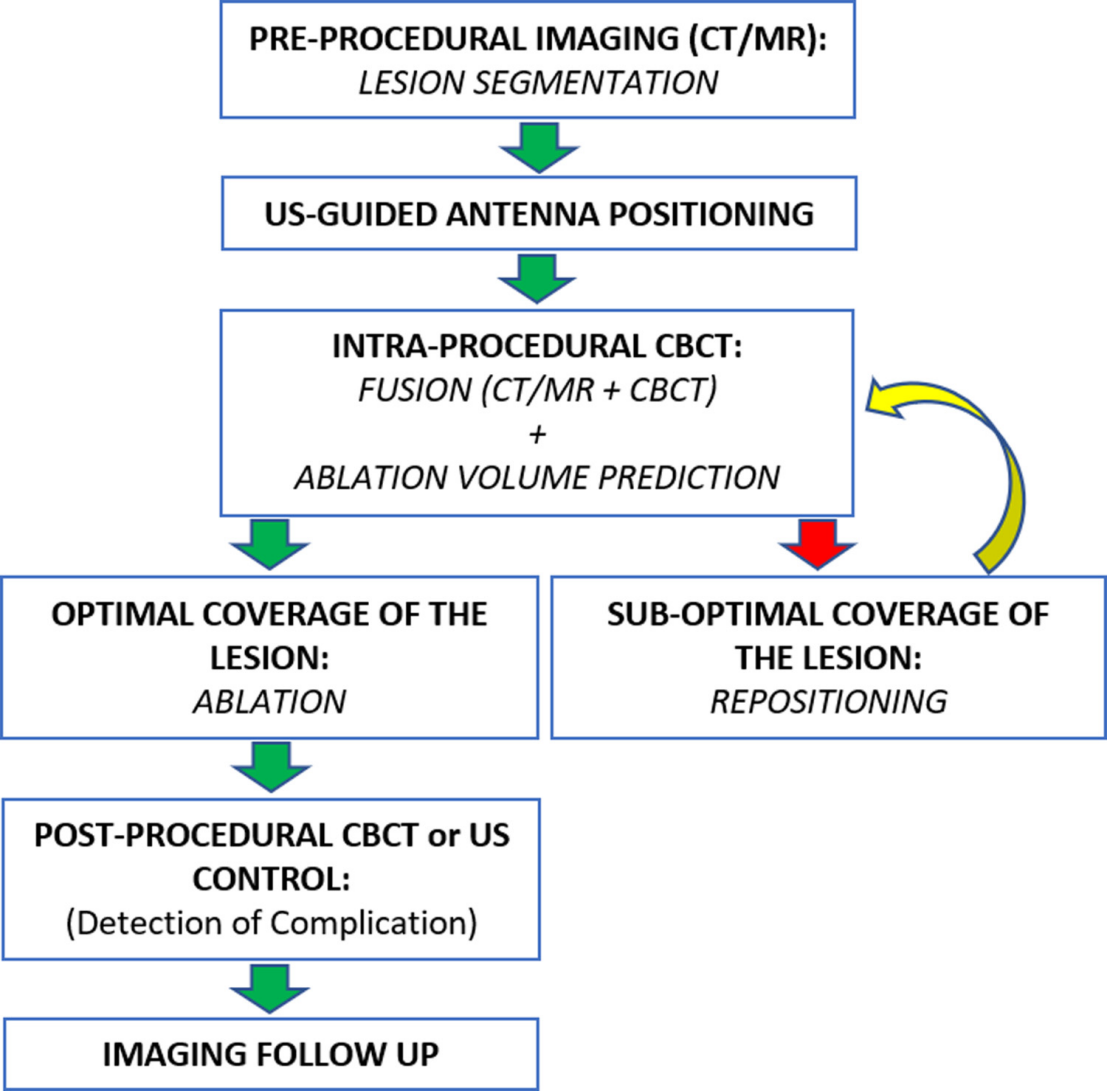
Schematic procedure flowchart.

A schematic flowchart of the procedure is resumed in Figure 3.

### Data Analysis

Data analysis were performed following the criteria of the “Consensus Guidelines for the Definition of Time-to-Event End Points in Image-guided Tumor Ablation.”^13^

We evaluated for each procedure or lesion: technical success, technique efficacy, time of the procedure, need for antenna reposition, residual unablated tumor, and complications.

*Technical success* addressed whether the tumor was treated according to protocol and covered completely by the ablation zone, by using Prediction Ablation Volume Software.

*Technique efficacy* referred to a prospectively defined point in time *(1 month FU)* when complete ablation of imageable target tumor was achieved, as evidenced by imaging FU (contrast CT or MRI) interpreted by an independent radiologist.

*Residual unablated tumor* referred to the presence of residual viable tumor at the ablative margin at initial FU imaging.

*Complications* were evaluated following the CIRSE standard.^27^

## Results

The MW antenna (13 gauge) was placed under US guidance in 25 tumors and with US-CT or US-MRI fusion guidance in 7 tumors. Technical success was achieved in 30 of the 32 treatment (94%). In 2 procedures the CBCT images didn’t allow the desired subjective quality of fusion-imaging between CBCT and CT/MRI. Average time of the procedure was 49 min (20-120 min). The antenna was repositioned (following placement but before ablation) in 16 of 30 (53%) of evaluable target lesions, wherein use of the flowchart (based upon prediction of the ablation volume software) resulted in repositioning of the antennae. We experienced 2 grade II (pain, vomiting managed with drug therapy, self-limiting hemoperitoneum) and 1 grade III CIRSE Classification System's complication (bleeding managed with embolization, without sequelae).^27^ We evaluated the FU contrast CT/MRI following LIRADS and RECIST criteria.^23,28^ Technical efficacy was evaluated in the population of 30 lesions in which we achieved technical success. In this group of lesions, there were 4 unablated residual tumors at the FU achieving a technical efficacy of 87% (26 of 30).

The study population is shown in Table 2.

**Table 2.** Study Population Data and Outcomes Summarized.

Patient	Lesion (number)	Volume of the lesion	Segment location	Tech. success	Antenna repositioning (time)	Intraprocedural CBCT number	Residual tumor 1 month FU	Tech. efficacy
1	HCC(1)	1.3	IV/V	Yes	Yes	3	No	Yes
2	HCC(1)	3.6	VIII	Yes	Yes	2	No	Yes
3	HCC(1)	3.2	V	Yes	No	1	No	Yes
4	CRC-METS(1)	3.5	VII	Yes	No	1	No	Yes
5	HCC-CC(1)	1.2	VI	Yes	Yes	2	No	Yes
6	HCC (1)	1.9	VI	Yes	Yes	2	No	Yes
7	HCC(1)	0.7	III	Yes	No	1	No	Yes
8	HCC(1)	1.8	VIII	Yes	Yes	3	No	Yes
9	HCC(1)	0.6	IV/VIII	Yes	No	1	No	Yes
10	HCC(1)	2.8	IVa	Yes	No	2	No	Yes
11	HCC(1)	4.7	V	Yes	Yes	3	No	Yes
12	CRC-MET(2)	2.7–2.1	V-VIII	Yes	Yes	2	No-No	Yes-Yes
13	HCC(1)	1.7	VI	No	No	2	No	-
14	HCC(1)	1.4	VIII	Yes	Yes	2	No	Yes
15	HCC(1)	1.1	V/VI	Yes	No	1	No	Yes
16	HCC(1)	1	VIII	Yes	Yes	3	Yes	No
17	HCC(1)	2.3	VI	Yes	No	1	No	Yes
18	HCC(1)	1.7	VI/VII	Yes	Yes	7	No	Yes
19	HCC(1)	1.8	V	No	No	1	No	Yes
20	HCC(1)	4	V/VI	Yes	Yes	5	No	Yes
21	HCC(1)	3.1	V	Yes	No	1	No	Yes
22	HCC(2)	2.5–1.6	VI-VIII	Yes	No	1	No-No	Yes-Yes
23	HCC(2)	1.4–0.8	VIII-VII	Yes	No	1	Yes-No	No-Yes
24	HCC(1)	2.5	VI	Yes	Yes	1	Yes	No
25	HCC(1)	2.2	V	Yes	Yes	2	No	Yes
26	HCC(1)	8.6	VIII	Yes	No	1	No	Yes
27	HCC(1)	0.7	VIII	Yes	Yes	1	No	Yes
28	HCC(1)	1	VII	Yes	Yes	2	No	Yes
29	HCC(1)	2.3	II/III	Yes	No	2	No	Yes
TOT: 29	32	-		30 of 32 (94%)	16 of 32 (50%)	-	3 of 32 (9%)	29 of 32 (91%)

Abbreviations: CRC, colorectal cancer; HCC, hepatocellular carcinoma; HCC-CC, hepatocholangiocarcinoma; TOT, total.

## Discussion

IO is becoming more impactful with increasing impact as higher level evidence is obtained for cancer therapies. The growing importance of percutaneous ablation is strictly linked to the development of new technologies and software helping interventional radiologists to perform more precise procedures achieving better outcomes, in a standardized and reproducible fashion. In situ ablation can risk residual unablated tumor and local recurrence when lesions are too large or difficult to see, or are in technically challenging locations. The link between local recurrence and the lack of an appropriate ablative margin has been well established.^29^ This may be linked to the presence of residual micrometastasis at the tumor margins or elsewhere in tumor rests that are not detectable with imaging modalities due to the small dimension or molecular characteristics.^30^ It is important to underline that despite other factors linked to local recurrence (like tumor histology or dimensions), margin ablation is one factor strictly linked to complete response that depends on the operator's technical skills and ability to target the tumor and verify the margin. This factor may be assisted with software and computer vision for planning, seeing, treating, and verifying the treatment. CIRSE standard of practice for liver ablation outlines the importance of an ablation margin of 5 to 10 mm beyond the tumor burden.^21^ Unfortunately, with traditional imaging guidance modalities (CT and US), it may not be easy to assess the exact tumor nor ablation margins. Despite that, in clinical practice most interventional radiologists evaluate the ablation subjectively by cognitively comparing pre-ablation and postablation imaging.^31,32^ The importance of evaluating the achievement of a proper complete ablation within the ablative margin is outlined also from the Consensus Guidelines from SIO and DATECAN. Moreover, 2 trials evaluating the concept of using dedicated software to evaluate the ablative margin are ongoing (Ablation Confirmation[NCT03753789] and Cover All Study [NCT04083378]), confirming the big interest of the scientific community in the implementation of ablation software to help IR achieving better and more reproducible and reliably standardized outcomes. However these trials are focused on CT-guided ablation. Since not all the interventional radiologists have access to a dedicated CT, in the “Ablation Confirmation Era” there may be needed an alternative available in every angio-suite. The CBCT Predictive Ablation Volume Software may be implemented as an additional/alternative tool for Ablation Confirmation (in addition to planning, navigation, and targeting). One of the main advantages of the use of this software is the possibility of assessing the ablation zone before turning on the probe, giving the operators the possibility of repositioning the probe to achieve a more precise treatment, and avoid the burn of nontarget tissue. This tool may be particularly useful when performing multiple-needle treatments, when the planning and evaluation of the ablation volume become even more challenging for the operator. In literature there are few previous papers evaluating, on small heterogenous populations, the application of prediction ablation software on CBCT.^26,33–35^ In our population and workflow, the probe placement was modified in 53% of the procedures. The use of the flowchart based upon prediction of the ablation volume software resulted in repositioning of the antennae in 16 of our treatments out of 30, strongly modifying the procedure workflow. Although speculative, this may have avoided the need of several retreatments or more treatment failures. The main difference of our flowchart from the one previously described by Floridi et al for the treatment of liver lesion is the use of a previous contrast CT/MRI instead of an enhanced CBCT for the fusion imaging. The lack of requiring an enhanced CBCT preprocedure may have contributed to the lower average procedure time in our study (49 vs 62 min), while achieving similar technical success and efficacy.^26^ The use of a semi-automatic software as the one described from Solbiati et al,^34^ could help shorting operative time and obtain better fusion imaging quality, thanks to the automatic elastic registration. One of the disadvantages of this procedure flowchart may be linked to the longer operating time and the learning curve. On the other hand the possible lower need of reintervention could justify it. In 2 procedures, the patients’ respirate motion may have been a factor causing CBCT motion artifacts that didn’t allow us obtaining high-quality fusion imaging, causing technical failure. However the use of general anesthesia may help overcome this limit. The results obtained in our study, with a technical success of 94% and technical efficacy of 87% are encouraging and add new data to the growing literature focused on Ablation Confirmation, further defining the value of a CBCT Prediction Ablation Volume Software. A prospective study on a larger population and with longer FU is needed to further assess the clinical efficacy of this technique.

Our study presents several limitations. First of all despite is one of the largest cohort of patient treated using the Prediction Ablation Volume Software, it is a retrospective and nonconsecutive study, that introduce limitations and bias since the study is an assessment of experienced users only. Prospective larger study with longer FU would be more convincing.

We didn’t perform any calculation or justification for the sample size. This study design does not specifically show the cause and effect of the probe manipulations based on software. We included patients with different histologies, which causes sample inhomogeneities. Lastly, prediction of the ablation compared to postablation assessment is weakened by potential technical variability of ablations, which do not always recapitulate the manufacturers expected ablation dimensions due to several factors. A postablation contrast scan could be compared to the expected treatment volumes to assess this variability in future studies.

## Conclusions

In conclusion, the implementation of a CBCT Prediction Ablation Volume Software and flowchart for the treatment of liver malignancies altered the procedure, and the approach demonstrated high technical success and efficacy. Fusion may be useful for guidance (often with US fusion) and CBCT fusion may be useful for verification purposes in the same patient. Such tools should be useful procedural prediction, implementation and verification of ablation. It may potentially be evaluated as an additional tool for ablation confirmation in liver percutaneous ablation treatments, to help interventional radiologists achieve more precise and tailored treatments.

## Supplemental Material

sj-docx-1-tct-10.1177_15330338231181284 - Supplemental material for Virtual Treatment Zone From Cone Beam CT Commonly Alters Treatment Plan and Identifies Tumor at Risk for Under-Treatment in US or US Fusion-Guided Microwave Ablation of Liver Tumors Click here for additional data file. Supplemental material, sj-docx-1-tct-10.1177_15330338231181284 for Virtual Treatment Zone From Cone Beam CT Commonly Alters Treatment Plan and Identifies Tumor at Risk for Under-Treatment in US or US Fusion-Guided Microwave Ablation of Liver Tumors by Antonio Arrichiello, Anna Maria Ierardi, Alessandro Caruso, Pasquale Grillo, Letizia Di Meglio, Pierpaolo Biondetti, Massimo Iavarone, Angelo Sangiovanni, Salvatore Alessio Angileri, Chiara Floridi, Bradford Wood and Gianpaolo Carrafiello in Technology in Cancer Research & Treatment
