# Supplementary material for: Virtual Treatment Zone From Cone Beam CT Commonly Alters Treatment Plan and Identifies Tumor at Risk for Under-Treatment in US or US Fusion-Guided Microwave Ablation of Liver Tumors
Source: Technol Cancer Res Treat. 2023 Aug 22;22:15330338231181284. doi: 10.1177/15330338231181284 (PMC10467384; doi:10.1177/15330338231181284)
Supplement: sj-docx-1-tct-10.1177_15330338231181284 - Supplemental material for Virtual Treatment Zone From Cone Beam CT Commonly Alters Treatment Plan and Identifies Tumor at Risk for Under-Treatment in US or US Fusion-Guided Microwave Ablation of Liver Tumors [file sj-docx-1-tct-10.1177_15330338231181284.docx]

Supplementary Tables

| **Patient** | **Lesion** (number) | **Diagnosis** | **Aetiology** | **BCLC** | **CPT** | **MELD** | **ECOG** |
| --- | --- | --- | --- | --- | --- | --- | --- |
| 1 | HCC(1) | Lirads 4 | Primary Biliary Cirrhosis | A | A | 7 | 0 |
| 2 | HCC(1) | Lirads 4 | Alcoholic Liver Disease | A | A | 7 | 0 |
| 3 | HCC(1) | Histology | NASH | A | A | 7 | 0 |
| 4 | CRC-METS(1) | Histology | Mets from Colon Cancer | - | - | - | 0 |
| 5 | HCC-CC(1) | Histology | HBV Hepatitis | A | A | 7 | 0 |
| 6 | HCC (1) | Lirads 4 | HCV Hepatitis | A | A | 9 | 0 |
| 7 | HCC(1) | Histology | HCV Hepatitis | A | A | 7 | 0 |
| 8 | HCC(1) | Lirads 5 | HCV Hepatitis | A | A | 7 | 0 |
| 9 | HCC(1) | Lirads 4 | HCV Hepatitis | A | A | 8 | 0 |
| 10 | HCC(1) | Lirads 4 | HCV Hepatitis | A | A | 7 | 0 |
| 11 | HCC(1) | Lirads 5 | HBV Hepatitis | 0 | A | 6 | 0 |
| 12 | CRC-MET(2) | Histology | Mets from  Colon Cancer | - | - | - | 0 |
| 13 | HCC(1) | Lirads 4 | HBV Hepatitis | A | A | 6 | 0 |
| 14 | HCC(1) | Lirads 4 | HCV Hepatitis | A | B | - | 0 |
| 15 | HCC(1) | Lirads 4 | NASH | A | A | 9 | 0 |
| 16 | HCC(1) | Lirads 4 | Alcoholic Liver Disease | A | B | 13 | 0 |
| 17 | HCC(1) | Lirads 5 | HCV Hepatitis | A | A | 7 | 0 |
| 18 | HCC(1) | Lirads 5 | HBV Hepatitis | A | A | 9 | 0 |
| 19 | HCC(1) | Lirads 4 | HCV Hepatitis | 0 | A | 7 | 0 |
| 20 | HCC(1) | Lirads 5 | Alcoholic Liver Disease | A | B | 15 | 0 |
| 21 | HCC(1) | Lirads 4 | NASH | A | A | 8 | 0 |
| 22 | HCC(2) | Lirads 4 | HCV Hepatitis | A | A | 8 | 0 |
| 23 | HCC(2) | Lirads 4 | HCV Hepatitis | A | B | 7 | 0 |
| 24 | HCC(1) | Lirads 4 | HCV Hepatitis | A | A | 7 | 0 |
| 25 | HCC(1) | Lirads 4 | Alcoholic Liver Disease | A | A | 7 | 0 |
| 26 | HCC(1) | Lirads 5 | HCV Hepatitis | A | A | 7 | 0 |
| 27 | HCC(1) | Lirads 4 | HCV Hepatitis | A | A | 10 | 0 |
| 28 | HCC(1) | Lirads 4 | Alcoholic Liver Disease | A | A | 7 | 0 |
| 29 | HCC(1) | Lirads 4 | HCV Hepatitis | A | A | 7 | 0 |

**Table a**: Patients characteristics.

| **Patient** | **Lesion** (number) | **Volume**  **Of the**  **Lesion** | **Segment Location** | **Ablation  Power** (Watt) | **Ablation Time**  (min) |
| --- | --- | --- | --- | --- | --- |
| 1 | HCC(1) | 1.3 | IV/V | 100 W | 4 |
| 2 | HCC(1) | 3.6 | VIII | 100 W | 3 |
| 3 | HCC(1) | 3.2 | V | 100 W | 3 |
| 4 | CRC-METS(1) | 3.5 | VII | 100 W | 5 |
| 5 | HCC-CC(1) | 1.2 | VI | 100 W | 5 |
| 6 | HCC (1) | 1.9 | VI | 100 W | 4 |
| 7 | HCC(1) | 0.7 | III | 100 W | 3 |
| 8 | HCC(1) | 1.8 | VIII | 100 W | 3 |
| 9 | HCC(1) | 0.6 | IV/VIII | 100 W | 3 |
| 10 | HCC(1) | 2.8 | IVa | 100 W | 3 |
| 11 | HCC(1) | 4.7 | V | 100 W | 3 |
| 12 | CRC-MET(2) | 2.7-2.1 | V-VIII | 100 W | 3-4 |
| 13 | HCC(1) | 1.7 | VI | 100 W | 4 |
| 14 | HCC(1) | 1.4 | VIII | 100 W | 5 |
| 15 | HCC(1) | 1.1 | V/VI | 100 W | 5 |
| 16 | HCC(1) | 1 | VIII | 100 W | 4 |
| 17 | HCC(1) | 2.3 | VI | 100 W | 4 |
| 18 | HCC(1) | 1.7 | VI/VII | 100 W | 5 |
| 19 | HCC(1) | 1.8 | V | 100 W | 4 |
| 20 | HCC(1) | 4 | V/VI | 100 W | 5 |
| 21 | HCC(1) | 3.1 | V | 100 W | 4 |
| 22 | HCC(2) | 2.5-1.6 | VI-VIII | 100 W | 4-3 |
| 23 | HCC(2) | 1.4-0.8 | VIII-VII | 100 W | 4-3 |
| 24 | HCC(1) | 2.5 | VI | 100 W | 4 |
| 25 | HCC(1) | 2.2 | V | 100 W | 5 |
| 26 | HCC(1) | 8.6 | VIII | 100 W | 10 |
| 27 | HCC(1) | 0.7 | VIII | 100 W | 5 |
| 28 | HCC(1) | 1 | VII | 100 W | 3 |
| 29 | HCC(1) | 2.3 | II/III | 100 W | 3 |

**Table b**: Ablation Protocol details.
